# Surgical Instruments

**Published:** 1881-07

**Authors:** 


Surgical Instruments.—The profession at this end of the State have long felt the need of a reliable surgical instrument house in this city, where they could promptly obtain any instrument required at as low prices as New York houses offered them. We therefore gladly draw attention to the enterprise of C. M. Lyman, (Mr. Peabody's successor) in so improving his stock of instruments, that he is able to compete in prices with the largest houses in the country. On making a recent purchase we were agreeably astonished by a discount from catalogue price of twenty-five per cent., which Mr. Jeffrey, the polite and attentive manager of this department, informs us is now the regular discount.

